# Paraxanthine and azilsartan attenuate gentamicin-induced renal fibrosis *via* modulation of TGF-β1/Smad3/7 signaling and miRNA-21/miRNA-200b expression

**DOI:** 10.1186/s12967-026-08051-y

**Published:** 2026-05-11

**Authors:** Nany Saad Rizk El-Adwy, Nahla E. El-Ashmawy, Ghada M. Al-Ashmawy, Naglaa F. Khedr

**Affiliations:** 1 Department of Biochemistry, Faculty of Pharmacy, Tanta University, El-Geish Street, Medical Campus, Tanta, 31527 Egypt; 2 Pharmacist in El-Nasr Hospital, Port Said Governorate, Port Said, Egypt; 3 Department of Pharmacology and Biochemistry, Faculty of Pharmacy, The British University in Egypt, El Sherouk City, 11837 Egypt; 4 Department of Biochemistry, Faculty of Pharmacy, Alsalam University in Egypt, Kafr El Zayat, Egypt

**Keywords:** Azilsartan, Gentamicin, Renal fibrosis, miRNA-21/miRNA-200b, Paraxanthine, TGF-β_1_/Smad signaling

## Abstract

**Background:**

Renal fibrosis is a key contributor to chronic kidney disease progression. The TGF-β1/Smad signaling pathway, particularly Smad2 and Smad3 mediate pro-fibrotic responses, while Smad7 exerts inhibitory effects.

**Aim:**

The study aimed to evaluate the therapeutic potential of paraxanthine (Para), an active caffeine metabolite, and azilsartan (Azil), an angiotensin II receptor blocker, in attenuating gentamicin (GM)-induced renal fibrosis through targeting Smad pathway and miRNA-21/200b.

**Methods:**

Seventy male albino mice were randomized into seven groups (*n* = 10): Control, GM, SIS3 + GM, Para + GM, Azil + GM, Para + SIS3 + GM, and Azil + SIS3 + GM. GM (40 mg/kg, I.P.) was administered daily for 7 days. Subsequently, Para (20.5 mg/kg) and Azil (5 mg/kg) were given orally, while SIS3 (2 mg/kg, I.P.) was administered for 7 days. Serum and urine renal function markers were measured. Renal histopathology, protein expression of TGF-β1, CTGF, Smad3, Smad2 and Smad7, and gene expression of miRNA-21 and miRNA-200b were evaluated.

**Results:**

GM caused significant (*p* < 0.001) nephrotoxicity with elevated BUN, serum creatinine, urinary albumin/creatinine ratio, and KIM-1 and increased fibrosis and marked type I collagen deposition compared to normal control. However, treatments with Para & Azil resulted in significant (*p* < 0.05) improvement in renal functions. Pro-fibrotic markers TGF-β1, connective tissue growth factor (CTGF), Type I Collagen, Smad 2 and Smad3 were reduced, while Smad7 was increased in treated groups *versus* GM group. Additionally, miRNA-21 was downregulated and miRNA-200b was upregulated following treatments.

**Conclusion:**

Paraxanthine and azilsartan demonstrated significantl restoring of kidney function and suppressing fibrotic progression. Their actions were mediated through regulation of Smad3/7 signaling and modulation of miRNA-21 and miRNA-200b expression, highlighting these pathways as promising therapeutic targets for the treatment of renal fibrosis.

**Supplementary information:**

The online version contains supplementary material available at 10.1186/s12967-026-08051-y.

## Introduction

Kidney fibrosis is a progressive and debilitating condition characterized by excessive accumulation of extracellular matrix such as collagen and fibronectin within the kidney parenchyma. This process results in the replacement of functional nephrons with scar tissue (glomerulosclerosis and tubulointerstitial fibrosis) leading to impaired kidney function and ultimately renal failure. This pathological process involves complex signaling pathways and cellular interactions, making it a challenging target for therapeutic intervention [1].

Gentamicin (GM), a vital aminoglycoside used extensively since 1963 to treat severe Gram-negative bacterial infections, is known for its nephrotoxic effects in animal and clincal studies. GM-induced nephrotoxicity involves the generation of reactive oxygen species (ROS) in vascular, glomerular, and tubular tissues, leading to reduced antioxidant defenses. GM nephrotoxicity is characterized by acute tubular necrosis, apoptosis, overexpression of TGF-β_1_, intracellular edema, elevated endothelin I, monocyte/macrophage infiltration, basal membrane disruption, and glomerular congestion [2, 3].

TGF-β family members activate Smad-mediated pathways, which are derived from the fusion of Caenorhabditis elegans Sma genes and Drosophila Mad—Mothers against decapentaplegic—genes [4]. The Smad family consists of eight Smad proteins divided into three unique subgroups: receptor-activated Smads (R-Smads: Smad1, Smad2, Smad3, Smad5, and Smad8), the common Smad (C-Smad4), and inhibitory Smads (I-Smads: Smad6 and Smad7) [5, 6]. The two crucial downstream mediators of canonical TGF-β signaling, which is highly active in fibrotic kidneys, are Smad2 and Smad3 [1]. Smad2 is protective in fibrosis, while Smad3 is detrimental [7]. Smad4 translocates into the nucleus after activation and regulates downstream target gene expression by forming a heteromeric complex with Smad2/3 [8]. However, Smad7, a negative regulator, exerts anti-inflammatory and anti-fibrotic effects [9]. In chronic kidney disorders, TGF-β_1_ and angiotensin II can induce Smad7 mRNA production, while activating Smad Ubiquitination Regulatory Factors (Smurfs) and Arkadia-dependent ubiquitin-proteasome pathways that result in post-transcriptional modification and degradation of Smad7 protein [10].

miRNAs are small (18–25 nucleotides), non-coding single-stranded RNAs that play an important role in different biological processes through the control of gene expression [11]. Several miRNAs were shown to play a role in the genesis and progression of renal disorders [12]. TGF-β_1_ activation increases miRNA-21 expression through Smad signaling [13]. McClelland et al., 2015 [14] linked the high miRNA-21 levels in diabetic nephropathy (DN) patients with increased fibrosis and worsening of kidney function. In rat proximal tubular epithelial cells, TGF-β_1_ induced miRNA-21 expression through Smad3 and Akt signaling by suppressing Smad7. On the other hand, miRNA-200 may exert antifibrotic effects by blocking epithelial-mesenchymal transition (EMT) in proximal tubular epithelial cells [15] and normal human kidney (HK-2) cells [16].

Paraxanthine is a primary metabolite of caffeine in humans. It acts as a central neurological stimulant by exhibiting higher binding affinities for adenosine A_1_ and A_2A_ receptors [17, 18]. Paraxanthine is also identified as the most effective inhibitor of production of connective tissue growth factor (CTGF), which was suggested as a therapeutic target for managing fibrotic diseases [19]. Preclinical in vitro and in vivo studies showed that paraxanthine has a better safety profile than caffeine and has a low abuse liability [20, 21].

Azilsartan, an angiotensin II type 1 (AT1) receptor blocker (ARB), exhibited nephroprotective effects by reducing inflammation, apoptosis, and oxidative stress, and antifibrotic effect by blocking the High Mobility Group Box 1 HMGB1/NF-κB/p38/ERK1/2/JNK signaling cascade in animal model [22]. Other Animal studies showed that azilsartan successfully decreased blood pressure, reduced cardiac hypertrophy, and protected against kidney injury. It has been reported to boost the levels of the beneficial peptide angiotensin-1–7 (Ang 1–7) and lower the detrimental eicosanoids such as 20-hydroxyeicosatetraenoic acid (20-HETE) [23].

The present study aimed to examine the antifibrotic effects of paraxanthine and azilsartan in GM-induced renal fibrosis in mice and to illustrate the underlying molecular mechanisms specifically regarding the involvement of TGF-β_1_/Smad pathway. Whether the expression of miRNA200b and miRNA-21 could be modulated by the effect of treatment was also evaluated.

## Materials and methods

### Drugs

Gentamicin^®^ with purity 98%-99%, was procured from MEMPHIS, El-Amirya Company, Egypt and was dissolved in 0.9% normal saline. The Smad3 inhibitor SIS3 (CAS No. 1009104-85-1) was purchased from BOCSCI Inc. (NY, USA) and was dissolved in 5% dimethyl sulfoxide. Paraxanthine (CAS No. 611–59-6) was purchased from BOCSCI Inc. (NY, USA) and was dissolved in carboxy methyl cellulose (CMC) (0.5% w/v). Azilsartan (CAS No. 147403-03-0) was obtained from Lihue Pharm Technology Co., Ltd., China. and was dissolved in CMC (0.5% w/v).

### Animals and experimental design

The study was approved by the Ethical Committee of the Faculty of Pharmacy, Tanta University, Egypt (Code: TP/RE/6/24 M-001). Male Albino mice, weighing 32-38 g, were obtained from Giza Institute of Ophthalmology (Cairo, Egypt). Animals were placed in metal cages at constant environmental conditions; 25 ± 2 °C with free access to water and animal chow for 2 weeks for acclimatzation. Then, seventy mice were randomly divided into seven groups; *n* = 10/group as follows: Control, GM, SIS3+GM, Para+GM, Azil+GM, Para+SIS3+GM and Azil+SIS3+GM. Gentamicin was injected I.P. at a dose of 40 mg/kg for 7 consecutive days [24]. GM was selected because it is a well-established and widely accepted experimental model of nephrotoxicity and renal fibrosis [2, 3]. Para was administered orally at a dose of 20.5 mg/kg [17], Azil was administered orally at a dose of 5 mg/kg [25] and SIS3 was injected I.P. at a dose of 2 mg/kg, half hour before oral administration of Para or Azil [26]. Para, Azil and SIS3 were given to mice daily for 7 days starting on day 8.

### Sample collection

At the end of experiment (on the 15^th^ day), each mouse was placed in a metabolic cage for 24-hour for urine collection. The collected urine samples were centrifuged at 765×g for 10 min and stored at −80 °C. After that, mice were fasted over night, weighed and anesthetized with isoflurane. Blood was obtained *via* cardiac puncture, serum was separated at 800×g for 20 min, and stored at −80 °C for biochemical analysis. Then, mice were euthanized by cervical dislocation, kidneys were immediately dissected, washed with normal saline, dried on paper towel, and weighed. The right kidneys were preserved in 10% buffered neutral formalin for histopathological analysis, while the left kidneys were stored at −80 °C for subsequent protein and gene expression analysis.

### Kidney index

The kidney index was calculated using the formula: Renal index (mg/g) = Kidney weight/body weight [27].

### Measurement of kidney function

Blood urea nitrogen (BUN) levels were measured colorimetrically using the urease-Berthlot technique [28] using commercial kits (Biodiagnostics, Giza, Egypt). Serum and urine creatinine levels were quantified colorimetrically according to the method described by Slot [29], using kits purchased from Biodiagnostic Co., Giza, Egypt and the concentration was calculated in mg/dL. Urinary albumin in 24 h urine sample was assessed colorimetrically using the bromocresol green method [30], using kits purchased from Biodiagnostic Co., Giza, Egypt. Urinary albumin-to-creatinine ratio (ACR) was calculated by dividing albumin concentration (mg/mL) by creatinine concentration (mg/mL) for each sample [31]. Urinary kidney injury molecule-1 (KIM-1) levels were measured by ELISA technique, using kits purchased from Sunred, Shanghai, China (Cat #201–11-0550); the sensitivity range was 23.77 pg/mL and the assay range 25–7200 pg/mL.

### Determination of kidney Smad2 concentration

Total Smad2 levels were quantified in kidney tissue homogenate using a commercially available rat Smad2 sandwich ELISA kit (Cat # orb781514) purchazed from Biorbyt Ltd., Cambridge, UK. Kidney tissue samples were homogenized in phosphate buffer (pH 7, 1:9 w/v), and the homogenate was centrifuged using Megafuge 16 R Centrifuge (Thermo Scientific, Germany) at 5000 ×g for 10 min and the supernatnent was used for analysis according to the manufacturer protocol. The sensitivity range was 0.058 ng/mL and detection range 0.16–10 ng/mL

### Western blot analysis of Smad3 and Smad7

Protein extraction was performed using the ReadyPrep™ Kit (Bio-Rad Inc, Cat #163–2086), and protein concentration was measured *via* the Bradford assay [32]. Samples (20 µg) were mixed with Laemmli buffer, boiled at 95 °C for 5 minutes, and subjected to SDS-PAGE using TGX Stain-Free™ gels. Proteins were transferred to a PVDF membrane using the Trans-Blot Turbo system. The membrane was blocked with tris-buffered saline containing tween 20 and 3% bovine serum albumin, incubated overnight at 4 °C with primary antibodies against Smad3 and Smad7 (1:500), and then washed. HRP-conjugated secondary antibody was applied for 1 hour, followed by chemiluminescent detection using the Clarity™ ECL substrate. Protein bands were analyzed using a CCD camera-based imaging system and normalized to β-actin.

### Quantitative reverse transcriptase polymerase chain reaction (qRT-PCR) of miRNA-21 and miRNA-200b

Total RNA was extracted from kidney tissue using the RNA-spin™ Total RNA Extraction Kit (iNtRON Biotechnology, Korea). RNA purity was confirmed with nanodrop spectrophotometer at 260/280 nm. One to five micrograms of total RNA were reverse-transcribed into complementary DNA (cDNA) using Thermoscript RNase Transcriptase (TIAGEN RT-PCR Kit, China). The cDNA was then analyzed by quantitative PCR using thermocycler (Thermo Fisher Scientific, PicoReal 5100, Finland). All primers were obtained from Biosearch Technologies Co. (USA) and the primers’ sequences [33, 34] are shown in Table 1.

**Table 1:** Sequences of primers of the target genes

Target gene	Primer sequence		Reference
miRNA-21	Forward	5’-CGGGATCCAGCCACTACCAAGGCATGTT-3’	[33]
	Reverse	5’-CGGAATTCAACCACGACTAGAGGCTGAC-3’	[33]
miRNA-200b	Forward	5’-GGGCTCCGCCGTCATCATTA-3’ by NCBI	NCBI
	Reverse	5’-GCCATCTTACTGGGCAGCATT-5’ by NCBI	NCBI
U6	Forward	5’-CTCGCTTCGGGACCACA-3’	[34]
	Reverse	5’-AACGCTTCAGGAATTTGGGT-3’	[34]

NCBI: National Centre for Biotechnology Information by Primer-Blast

The PCR protocol included 40 cycles with denaturation at 95 °C for 5 seconds and annealing/extension at 53 °C for 10 seconds. The relative copy number (RCN) of miRNA-21 or miRNA-200b was calculated and normalized to housekeeping gene U6 levels. Ct (threshold cycle) values were determined, and the 2^-ΔΔCt^ method was used to calculate relative gene expression [35].

### Histopathological examination of kidney tissue (H&E)

Kidney tissues were deparaffinized and rehydrated through a decreasing series of ethyl alcohol after being sectioned into 3-μm thick slices using a microtome (Leica RM 2135, Germany). The sections were then dried, cleared in xylene, and stained with hematoxylin and eosin (H&E). The stained slides were examined using light microscope (Olympus, Japan) by an expert pathologist who was blinded to the study design. Each kidney section was evaluated in at least six fields for tubular and glomerular structures. Abnormalities such as brush border loss, tubular degeneration, glomerular atrophy, and leukocytic infiltration were observed and scored based on severity: (-) not found, (+) mild, (++) moderate, and (+++) severe [36].

### Immunohistochemical analysis of CTGF and TGF-β_1_ in kidney tissue

Tissue sections from mice kidneys were deparaffinized and rehydrated, followed by blocking of endogenous peroxidase activity with hydrogen peroxide [37]. Antigen retrieval was performed using Santa’s target retrieval solution. The staining process involved incubating the samples with mouse Anti CTGF (Cat# sc 365970, RRID: AB_10917259) and mouse anti-human TGF-β_1_ (Cat #sc-130348, clone 3C11) antibodies from Santa Cruz Biotechnology, Inc, USA. CTGF expression was primarily observed in the cytoplasm with some membrane accentuation, while TGF-β_1_ expression was predominantly cytoplasmic [38]. All immunohistochemical (IHC) staining was counterstained with Mayer’s hematoxylin and imaged at magnifications of X100 (bar = 100 µm) and X400 (bar = 50 µm). Positive CTGF or TGF-β_1_ immunostaining is indicated by a brown tubular response and quantified as the percentage of brown color relative to the blue background using color image analysis equipment (Leica Qwin 500 image analyzer) coupled with Leica microscope, at a magnification of X400 [39].

### Histopathological examination of renal fibrosis by Sirius red staining

Picrosirius Red staining (Cat # 36-554-8) (Direct Red 80) from Sigma-Aldrich (Germany) was used to assess collagen deposition. After being deparaffinized, sections were rehydrated, stained for one hour with Picrosirius Red solution, cleaned with acidified water, dehydrated, clarified, and mounted [40, 41]. An experienced pathologist who was blind to the research design inspected the stained slides using a light microscope (Olympus, Japan).

### Immunohistochemical analysis of Collagen 1

Mouse kidney slices fixed in paraffin were deparaffinized and rehydrated using ethanol at several concentrations. Hydrogen peroxide incubation inhibited endogenous peroxidase activity [37]. Santa Cruz target retrieval solution was used for antigen retrieval in accordance with the manufacturer’s recommendations. A rabbit polyclonal anti-Collagen I antibody (Cat # GB115707) from Servicebio (Wuhan, China) was used to incubate sections with dilution 1:1000 for a whole night at 4 °C. As directed by the manufacturer, immunoreactivity was found utilizing the PolyVue Plus™ Mouse HRP as secondary antibody (Cat # PVP25D) and DAB detection system from Diagnostic BioSystems, USA. Mayer’s hematoxylin was used as a counterstain. After that, sections were cleaned, dehydrated, and mounted [42]. All immunohistochemical (IHC) staining was counterstained with Mayer’s hematoxylin and imaged at magnifications of X100 (bar = 100 µm) and X400 (bar = 50 µm). Positive brown interstitial staining for collagen 1 was quantified as the percentage of brown color using color image analysis equipment (Leica Qwin 500 image analyzer) coupled with Leica microscope, at a magnification of X400 [39].

### Statistical analysis

Statistical analysis was conducted using GraphPad Prism version 8.0.2. Data were presented as mean ± standard deviation (SD). One-way analysis of variance (ANOVA) followed by Tukey’s post hoc test for multiple comparisons was used to compare differences between groups. Statistical significance was defined at a *p*-value of less than 0.05. Data were analyzed for normality using normal (Gaussian) distribution test by Shapiro-Wilk method.

## Results

### Kidney index in the studied groups

Gentamicin induced nephrotoxicity was associated with significant increase in kidney index compared to control group (*p* < 0.05). However treated groups showed significant reduction in kidney index (*p* < 0.05) compared to GM, indicating that all treatments could protect the kidney against GM effect (Table 2).

**Table 2:** Kidney index in the studied mice groups

Groups	Kidney index (mg/g)
Control	7.08 ± 0.39
GM	15.55 ± 0.29^a^
SIS3+GM	8.49 ± 0.25^a,b^
Para+GM	8.47 ± 0.31^a,b^
Azil+GM	9.29 ± 0.19^a,b,c,d^
Para + SIS3 +GM	8.11 ± 0.08^a,b,e^
Azil + SIS3 +GM	8.83 ± 0.25^a,b,e,f^

Data are mean ± SD, significance was set at *p* < 0.05, *n* = 10. Gentamicin (GM): 40 mg/kg daily I.P. for 7 days. Paraxanthine (Para): 20.5 mg/kg orally daily for 7 days starting on day 8. Azilsartan (Azil): 5 mg/kg orally daily for 7 days starting on day 8. Smad3 inhibitor (SIS3): 2 mg/kg I.P. daily for 7 days starting on day 8. a: Significant vs control group, b: Significant vs GM group, c: Significant vs SIS3+GM, d: Significant vs Para+GM, e: Significant vs Azil+GM, f: Significant vs Para+SIS3+GM

### Blood urea nitrogen and serum creatinine in the studied groups

**Fig. 1:**
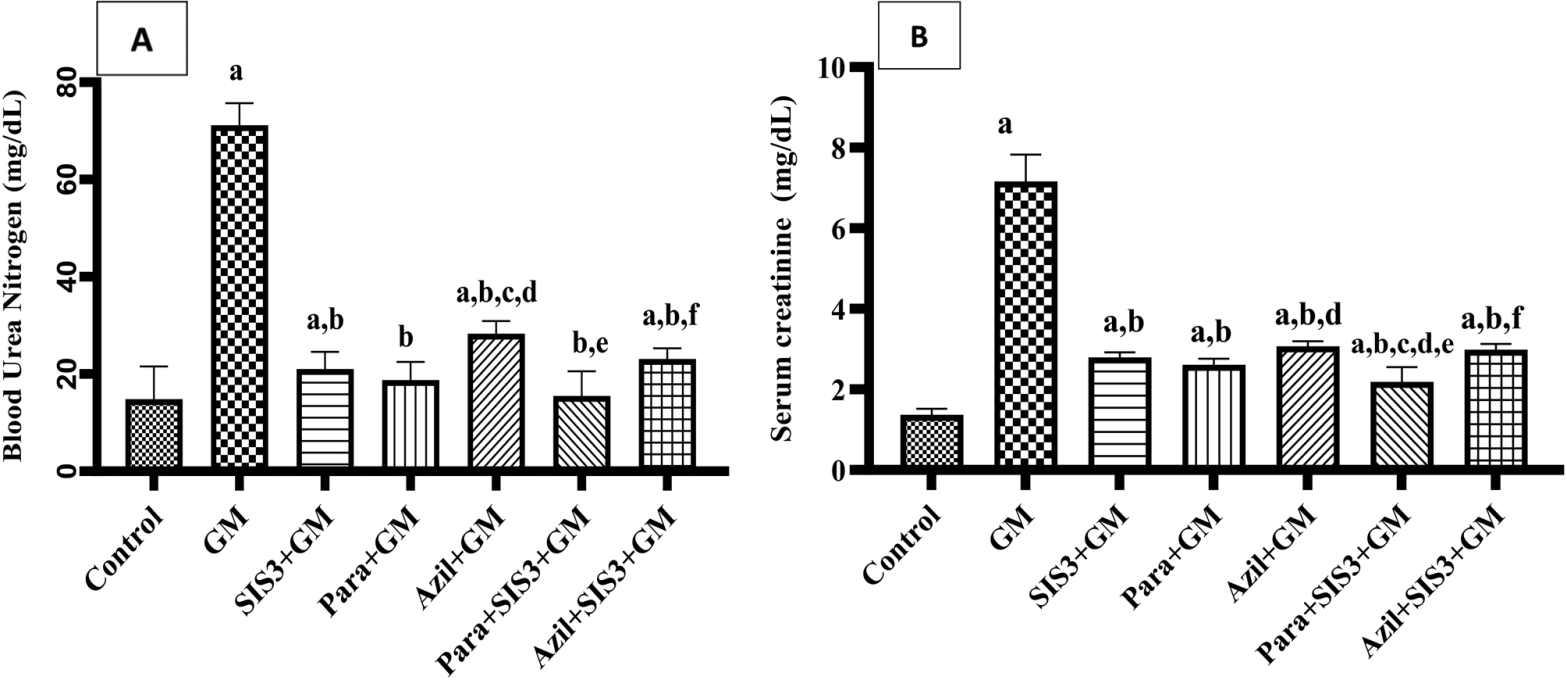
Effect of various treatments on (**A**): Blood urea nitrogen (BUN) and (**B**): Serum creatinine. Values are mean ± SD, One-way analysis of variance (ANOVA) followed by Tukey’s post hoc test for multiple comparisons was used to compare differences between groups, significance was set at *p* < 0.05, *n* = 10. Gentamicin (GM): 40 mg/kg daily I.P. for 7 days. Paraxanthine (Para): 20.5 mg/kg orally daily for 7 days starting on day 8. Azilsartan (Azil): 5 mg/kg orally daily for 7 days starting on day 8. Smad3 inhibitor (SIS3): 2 mg/kg I.P. daily for 7 days starting on day 8. a: Significant vs control group, b: Significant vs GM group, c: Significant vs SIS3+GM, d: Significant vs Para+GM, e: Significant vs Azil+GM, f: Significant vs Para+SIS3+GM

In GM group, BUN was significantly elevated compared to control group (71.15 ± 4.32 vs 14.31 ± 5.84 mg/dL, *p* < 0.05). All treated groups; SIS3+GM (21.02 ± 3.35 mg/dL), Para+GM (18.76 ± 3.46 mg/dL), Azil+GM (28.28 ± 2.25 mg/dL), Para+SIS3+GM (15.51 ± 4.82 mg/dL), and Azil+SIS3+GM (23.07 ± 2.09 mg/dL) showed significant reduction in BUN levels (*p* < 0.05) compared to GM group. However, the level of BUN in SIS3+GM (*p* = 0.01), Azil+GM (p<0.05) and Azil+SIS3+GM (p<0.05) groups remained significantly elevated comparable to those of normal control group (Fig. 1A).

Figure 1B showed that serum creatinine was significantly elevated in GM group compared to the control group (6.97 ± 0.64 vs 1.37 ± 0.14 mg/dL, *p* < 0.05). Serum creatinine in SIS3+GM (2.79 ± 0.12 mg/dL), Para+GM (2.61 ± 0.14 mg/dL), Azil+GM (3.07 ± 0.11 mg/dL), Para+SIS3+GM (2.18 ± 0.35 mg/dL), and Azil+SIS3+GM (2.99 ± 0.14 mg/dL) treated groups showed significant reduction (*p* < 0.05) compared to GM group. However, serum creatinine levels in all treated groups remained higher than that observed in the control group.

### Urinary kidney function markers in the studied groups

**Fig. 2:**
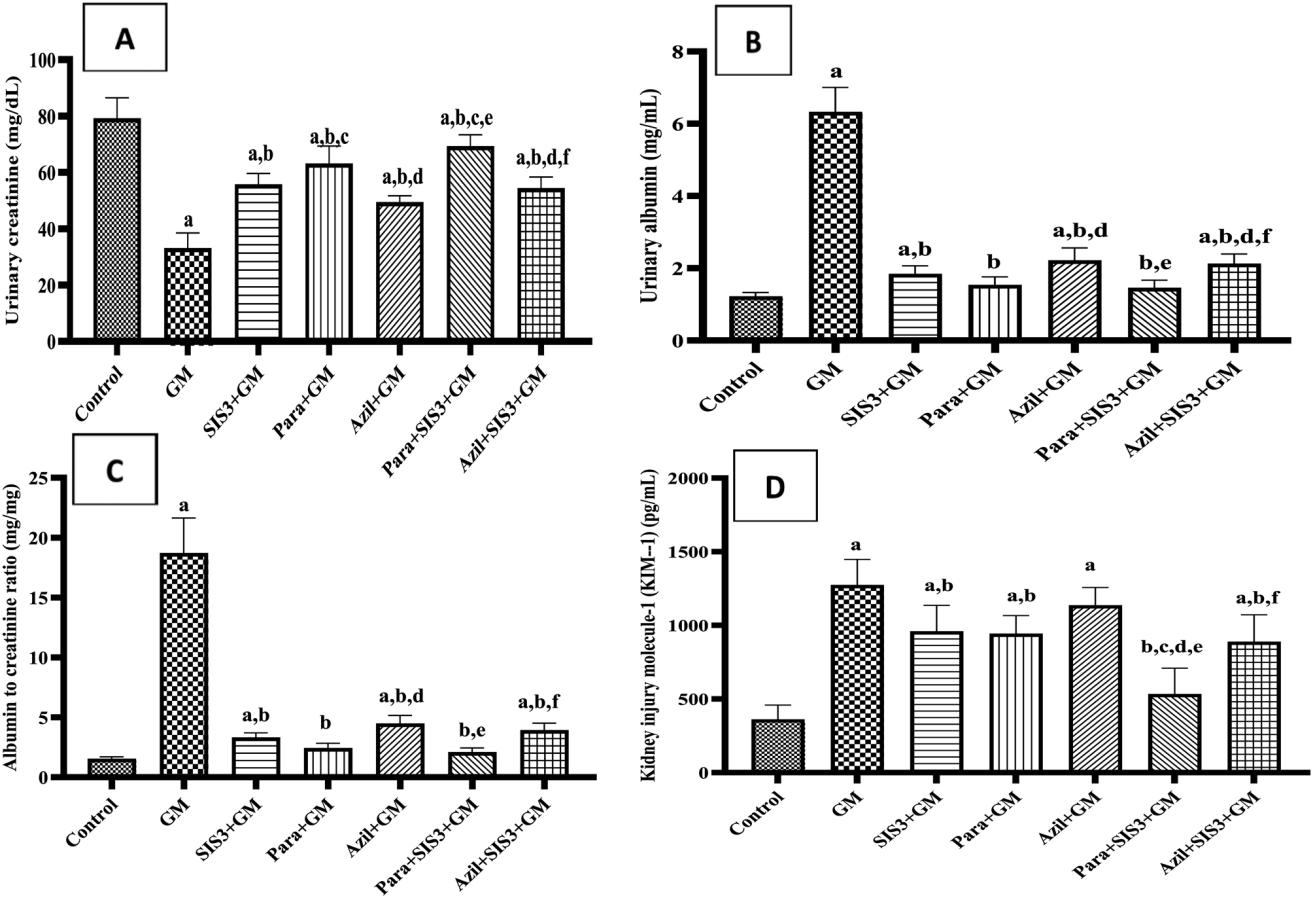
Effect of various treatments on (**A**): Urinary creatinine, (**B**): Urinary albumin, (**C**): Urinary albumin to creatinine ratio, (**D**): Urinary kidney injury molecule-1. Values are mean ± SD, One-way analysis of variance (ANOVA) followed by Tukey’s post hoc test for multiple comparisons was used to compare differences between groups significance was set at *p* < 0.05, *n* = 10. Gentamicin (GM): 40 mg/kg daily I.P. for 7 days. Paraxanthine (Para): 20.5 mg/kg orally daily for 7 days starting on day 8. Azilsartan (Azil): 5 mg/kg orally daily for 7 days starting on day 8. Smad3 inhibitor (SIS3): 2 mg/kg I.P. daily for 7 days starting on day 8. a: Significant vs control group, b: Significant vs GM group, c: Significant vs SIS3+GM, d: Significant vs Para+GM, e: Significant vs Azil+GM, f: Significant vs Para+SIS3+GM

GM group showed a significant decrease in urinary creatinine compared to control group (33.14 ± 5.04 vs 79.28 ± 6.81 mg/dL, *p* < 0.05) indicating renal damage. On the other hand, SIS3+GM (68.06 ± 3.60 mg/dL), Para+GM (63.19 ± 5.80 mg/dL), Azil+GM (49.43 ± 2.17 mg/dL), Para+SIS3+GM (69.37 ± 3.75 mg/dL), and Azil+SIS3+GM (54.43 ± 3.75 mg/dL) treated groups showed significant increase in urinary creatinine compared to GM group (p < 0.05) (Fig. 2A).

Figure 2B showed that GM group had significantly increased levels of urinary albumin compared to control group (6.33 ± 0.89 vs 1.23 ± 0.097 mg/mL, *p* < 0.05) indicating renal damage. However, urinary albumin levels were significantly decreased in SIS3+GM (1.85 ± 0.21 mg/mL), Para+GM (1.54 ± 0.21 mg/mL), Azil+GM (2.22 ± 0.32 mg/mL), Para+SIS3+GM (1.46 ± 0.20 mg/mL), and Azil+SIS3+GM (2.13 ± 0.25 mg/mL), (*p* < 0.05) compared to GM group. The least reduction in urinary albumin was obtained in Para+GM and Para+SIS3+GM groups, in which there was no significant difference vs control group.

Figure 2C showed that urinary albumin-to-creatinine ratio (ACR) was significantly increased in GM group compared to control group (18.72 ± 3.86 vs 1.55 ± 0.13 mg/mg, *p* < 0.05) indicating renal damage. On the contrary, ACR was significantly reduced in all treated groups, as follows; SIS3+GM (3.32 ± 0.36 mg/mg), Para+GM (2.45 ± 0.37 mg/mg), Azil+GM (4.49 ± 0.63 mg/mg), Para+SIS3+GM (2.11 ± 0.31 mg/mg), and Azil+SIS3+GM (3.94 ± 0.56 mg/mg), (*p* < 0.05) compared to GM group.

Figure 2D showed that GM group exhibited a significant increase in urinary KIM-1 level compared to control group (1274.17 ± 154.76 vs 361 ± 86.14 pg/mL, *p* < 0.05). However, all treatments showed significant (*p* < 0.05) reduction in KIM-1 level compared to GM group as follows; SIS3+GM (959.83 ± 157.08 pg/mL), Para+GM (944.17 ± 108.66 pg/mL), Azil+GM (1136.5 ± 107.67 pg/mL), Para+SIS3+GM (534.17 ± 155.65 pg/mL) and Azil+SIS3+GM (899.83 ± 161.88 pg/mL). Para+SIS3+GM treated group exhibited the most significant decrease in KIM-1 levels which was comparable to the control value, suggesting a superior protective effect against GM-induced kidney damage.

### Smad3/7 protein expression and total Smad2 concentration in the studied groups

**Fig. 3:**
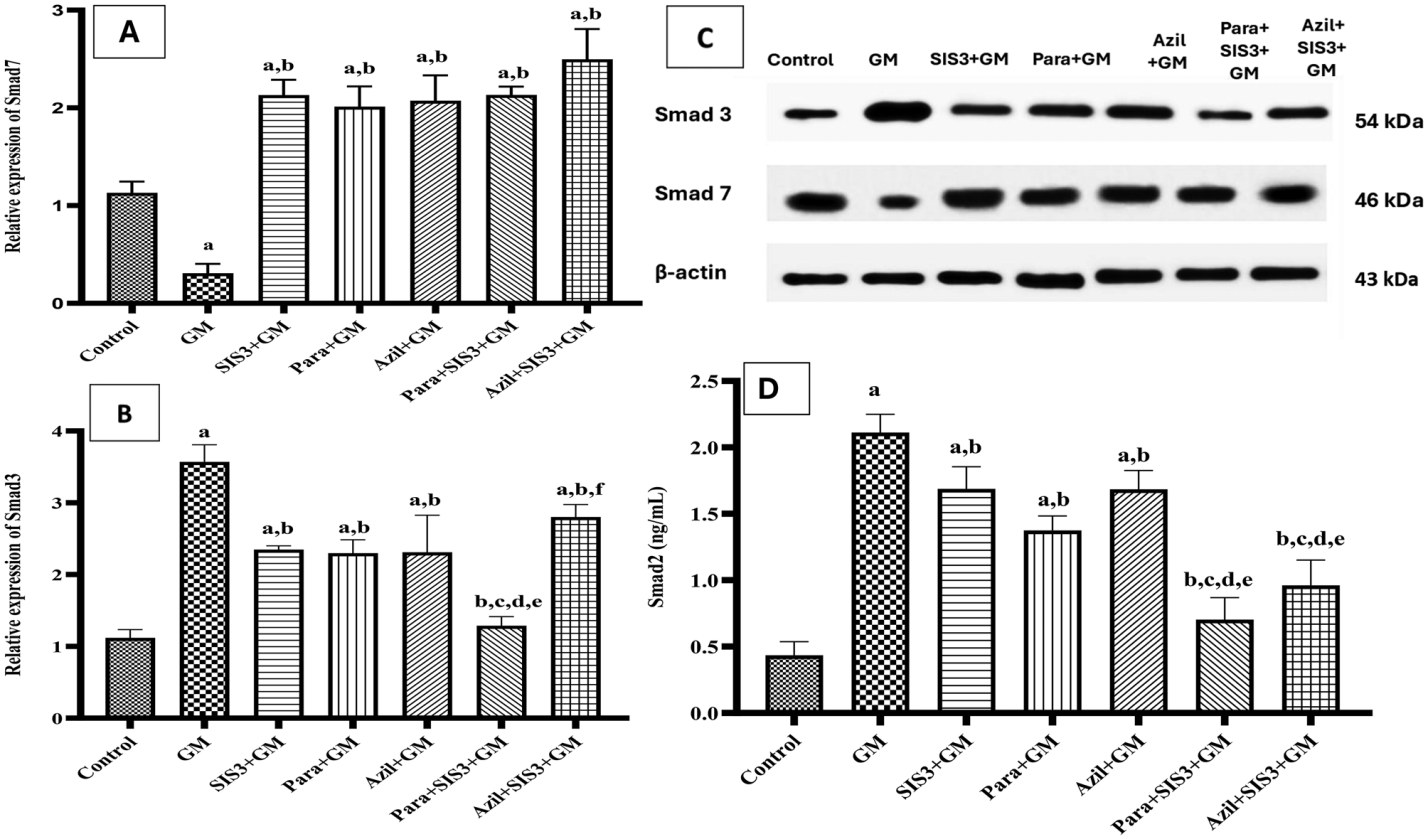
(**A**): Effect of various treatments on Smad7 relative protein expression, (**B**): Effect of various treatments on Smad3 relative protein expression, (**C**): Western blot graph of protein expression of Smad3 and Smad7 in the kidney of mice groups, (**D**): Effect of various treatments on kidney Smad2 protein concentration in mice groups. Values are mean ± SD, significance was set at *p* < 0.05, *n* = 3 (triplicate) for Western blots analysis, *n* = 10 for Sma2 analysis (ELISA technique). Gentamicin (GM): 40 mg/kg daily I.P. for 7 days. Paraxanthine (Para): 20.5 mg/kg orally daily for 7 days starting on day 8. Azilsartan (Azil): 5 mg/kg orally daily for 7 days starting on day 8. Smad3 inhibitor (SIS3): 2 mg/kg I.P. daily for 7 days starting on day 8. a: Significant vs control group, b: Significant vs GM group, c: Significant vs SIS3+GM, d: Significant vs Para+GM, e: Significant vs Azil+GM, f: Significant vs Para+SIS3+GM

Figure 3 A& C showed Western blot analysis of protein expression of Smad7. GM group showed a significant (*p* < 0.05) decrease in Smad7 protein expression compared to control group (0.31 ± 0.08 vs 1.13 ± 0.09 fold). However, SIS3+GM (2.13 ± 0.16), Para+GM (2.01 ± 0.17), Azil+GM (2.07 ± 0.21), Para+SIS3+GM (2.13 ± 0.07) and Azil+SIS3+GM (2.50 ± 0.25) treated groups showed significant increase in Smad7 levels, (*p* < 0.05) compared to GM group. Notably, Azil+SIS3+GM groups showed the highest Smad7 protein levels.

Figure 3B &C showed protein expression of Smad3 by Western blotting. GM group showed a significant (*p* < 0.05) increase in Smad3 protein expression compared to control group (3.57 ± 0.19 vs 1.12 ± 0.09 fold). However, SIS3+GM (2.35 ± 0.04), Para+GM (2.29 ± 0.19), Azil+GM (2.31 ± 0.42), Para+SIS3+GM (1.29 ± 0.10), and Azil+SIS3+GM (2.08 ± 0.14) treated groups showed a significant reduction in protein expression of Smad3 levels, (*p* < 0.05), compared to GM group. Notably, Para+SIS3+GM group exhibited the greatest reduction in Smad3 levels. Except for Para+SIS3+GM group, Smad3 level in all treated groups remained significantly higher than its value in the control group.

Figure 3D showed Smad2 concentration in kidney of studied mice groups. GM group showed a significant (*p* < 0.05) increase in Smad2 protein expression compared to control group (2.11 ± 0.14 vs 0.43 ± 0.10 ng/mL). However, SIS3+GM (1.68 ± 0.17), Para+GM (1.37 ± 0.11), Azil+GM (1.68 ± 0.14), Para+SIS3+GM (0.70 ± 0.17) and Azil+SIS3+GM (0.96 ± 0.19) treated groups showed significant decrease in Smad2 levels, (*p* < 0.05) compared to GM group. Notably, Para+GM, Para+SIS3+GM and Azil+SIS3+GM groups showed the lowest Smad2 concentrations among treated groups.

### Gene expression of miRNA-21 and miRNA-200b in the studied groups

**Fig. 4:**
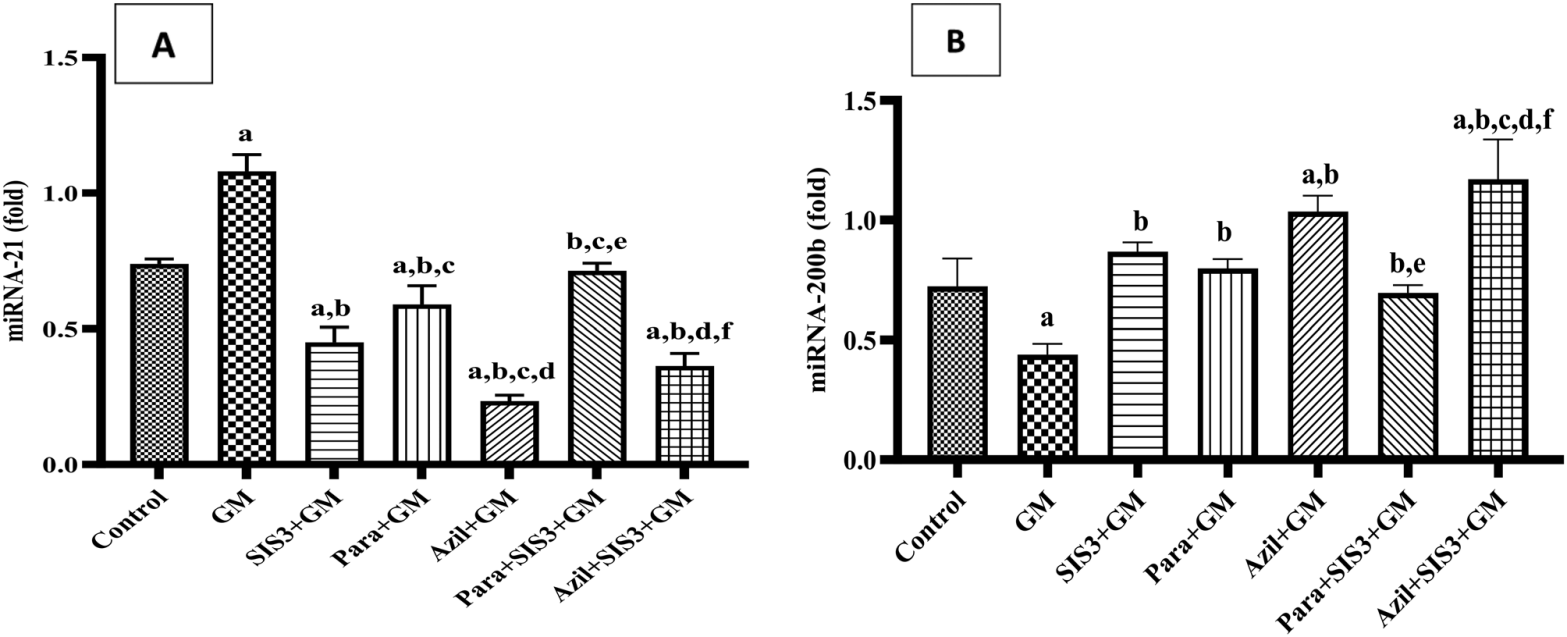
Effect of various treatments on gene expression of (**A**): miRNA-21 and (**B**): miRNA-200b. Values are mean ± SD, significance was set at *p* < 0.05, *n* = 3. Gentamicin (GM): 40 mg/kg daily I.P. for 7 days. Paraxanthine (Para): 20.5 mg/kg orally daily for 7 days starting on day 8. Azilsartan (Azil): 5 mg/kg orally daily for 7 days starting on day 8. Smad3 inhibitor (SIS3): 2 mg/kg I.P. daily for 7 days starting on day 8. a: Significant vs control group, b: Significant vs GM group, c: Significant vs SIS3+GM, d: Significant vs Para+GM, e: Significant vs Azil+GM, f: Significant vs Para+SIS3+GM

As shown in Fig. 4A, GM group showed a significant (*p* < 0.05) upregulation in miRNA-21 expression compared to control group (1.08 ± 0.05 vs 0.74 ± 0.02 fold). SIS3+GM (0.45 ± 0.04 fold), Para+GM (0.59 ± 0.05), Azil+GM (0.23 ± 0.02), Para+SIS3+GM (0.71 ± 0.02), and Azil+SIS3+GM (0.36 ± 0.04) treated groups demonstrated a significant downregulation in miRNA-21 expression (*p* < 0.05) compared to GM group. Except for Para+SIS3+GM group, all treated groups showed significantly lower expression of miRNA21 *versus* control group.

Figure 4B showed that GM group had a significant downregulation (*p* < 0.05) in miRNA-200b expression compared to control group (0.44 ± 0.03 vs 0.72 ± 0.09 fold). However, SIS3+GM (0.87 ± 0.03), Para+GM (0.79 ± 0.03), Azil+GM (1.03 ± 0.05), Para+SIS3+GM (0.69 ± 0.03), and Azil+SIS3+GM (1.17 ± 0.14) treated groups showed significant upregulation in miRNA-200b, (*p* < 0.05) compared to GM. Furthermore, treatments with Azil, either alone or in combination with SIS3, showed a significant increase in miRNA-200b expression compared to normal control group.

### Histopathological findings in the studied groups

**Fig. 5:**
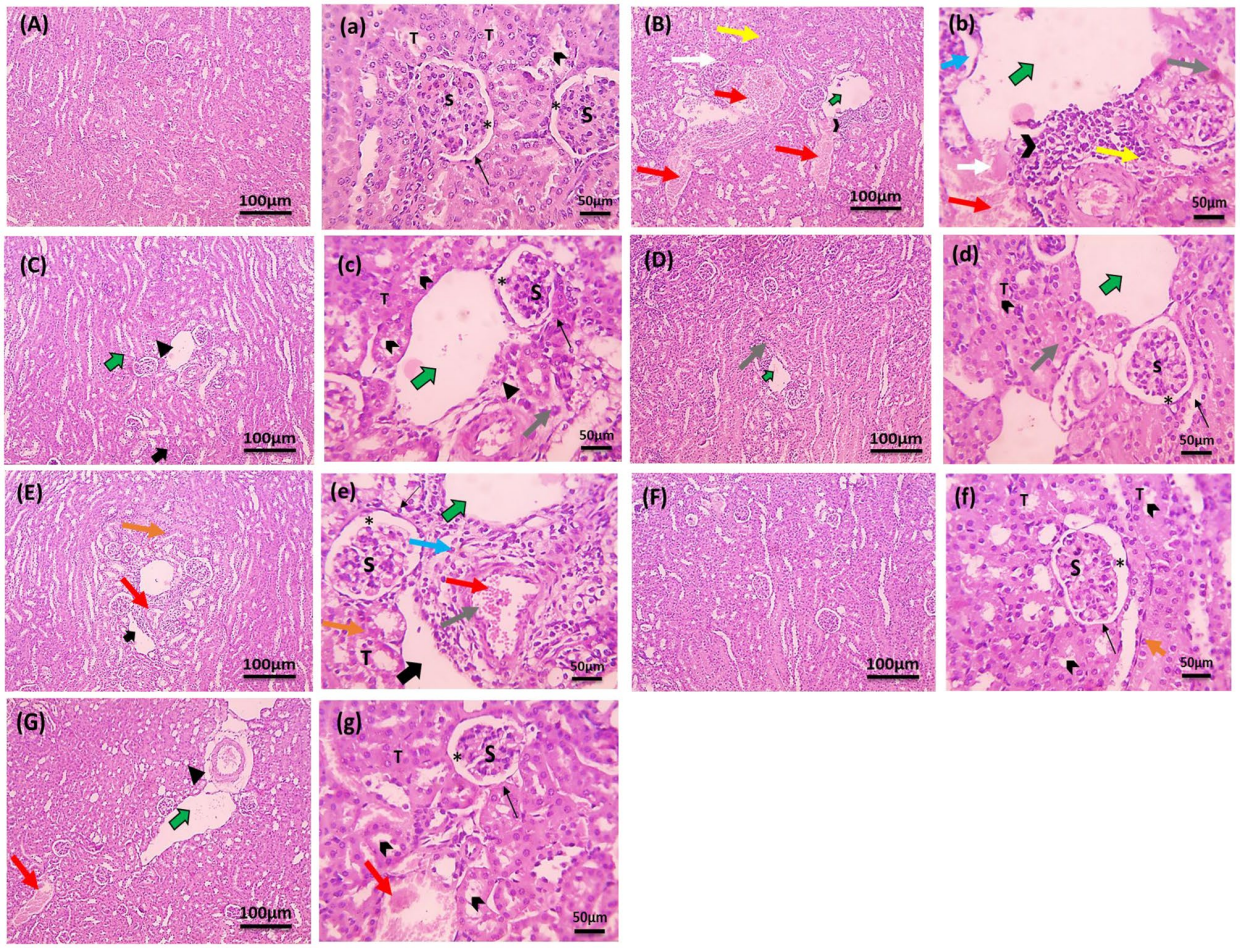
Photomicrographs of kidney tissues stained with H&E. Low magnification × 100 (large letters) and high magnification × 400 (small letters). (**A,a**) Section of normal control group showing normal glomerular corpuscles (S), Bowman’s capsule (thin black arrow), Bowman’s space (*) and normal tubules (T), having brush borders (small arrowheads) with minimal interstitial tissue. (**B,b**): GM group showing marked infiltration in interstitial tissue (opened arrowhead), fibrosis (blue arrow), edema (closed arrowhead), tubular epithelium suffering loss of brush borders, severely congested blood vessels (red arrow), dilated lymphatics (green arrow), leukocytic cells with hydropic degeneration (gray arrow) and coagulative necrosis (white arrow). The sections also showed areas of congestion (red arrow) and hemorrhage (yellow arrows). (**C,c**): SIS3+GM group displaying normal glomerular corpuscles (S), normal Bowman’s capsule (thin black arrow) with slightly dilated Bowman’s space (*) can be seen. The sections also showed normal tubules (T) that retained its brush borders (small arrowheads), mildly dilated lymphatics (green arrow), mild interstitial edema (closed arrowhead) with few leukocytic cells infiltration (gray arrow). (**D,d**): Para+GM group displaying normal kidney features with mildly dilated lymphatics (green arrow) and few leukocytic cells infiltration (gray arrow). (**E,e**): Azil+GM group displaying normal glomerular corpuscles (S), hypertrophy of epithelial lining Bowman’s capsule (thin black arrow) with dilated Bowman’s space (*), normal tubules (T) that retained its brush borders (small arrowheads), dilated lymphatics (green arrow), edema (closed arrowhead) with some leukocytic cells infiltration (gray arrow). The sections also revealed mild focal tubular hydropic degeneration (orange arrow), mildly congested blood vessel (red arrow), perivascular fibrosis (blue arrow) infiltrated with some leukocytic cells (opened arrowhead). (**F,f**): Para+SIS3+GM group displaying greatly improved histological picture with slightly dilated Bowman’s space (*) and normal tubules (T) that retained its brush borders (small arrowheads). However, few sections from this group showed mild hydropic degeneration in very few tubules (orange arrow). (**G,g**): Azil+ SIS3+GM group displaying normal glomerular corpuscles (S), normal Bowman’s capsule (arrow), normal tubules (T) that retained its brush borders (thin black arrowhead), moderately dilated lymphatics (green arrows), mildly congested blood vessel (red arrow) and perivascular edema (closed arrowhead)

Figure 5 showed kidney tissue sections stained with H & E from various experimental groups and Table (3) illustrated the scores of the histopathological lesions. Control group displayed normal glomerular corpuscles, Bowman’s capsule, and Bowman’s space. The tubules in this group exhibited well-preserved brush borders and minimal interstitial tissue, indicative of healthy renal tissue (Fig. 5A,a).

**Table 3:** Histopathological lesions in kidney tissues of studied groups

Histopathological lesions	Control	GM	SIS3+GM	Para+GM	Azil+GM	Para+SIS3+ GM	Azil+SIS3+GM
Loss of brush border	-	++	-	-	+	-	-
Tubular degeneration	-	++	-	-	++	-	++
Glomerular atrophy	-	++	-	-	-	-	-
Leukocytic infiltration	-	++	-	+	-	-	+
Congestion	-	+	-	-	+	-	-
Edema	-	+	-	-	+	-	+
Dilated lymphatics	-	++	-	-	+	-	-
Fibrosis	-	++	-	-	-	-	-
Hemorrhage	-	++	-	-	-	-	-

Data are presented as scores: (-) not found, (+) mild, (++) moderate, (+++) severe. *n* = 5. Gentamicin (GM): 40 mg/kg daily I.P. for 7 days. Paraxanthine (Para): 20.5 mg/kg orally daily for 7 days starting on day 8. Azilsartan (Azil): 5 mg/kg, orally daily for 7 days starting on day 8. Smad3 inhibitor (SIS3): 2 mg/kg I.P. daily for 7 days starting on day 8

In contrast, kidney tissue from GM group, revealed significant pathological changes; markedly congested blood vessels, dilated lymphatics, and infiltration of leukocytic cells into the interstitial tissue. The tubules demonstrated a loss of brush borders, severe hydropic degeneration, coagulative necrosis and areas of congestion and hemorrhage (Fig. 5B,b).

Figure 5C, c showed kidney tissue from the SIS3+GM group, which exhibited normal glomerular corpuscles, Bowman’s capsule, and Bowman’s space. The tubules had preserved their brush borders, with mildly dilated lymphatics and mild interstitial edema, accompanied by a few leukocytic cells. Figure 5D,d showed that in Para+GM group, normal glomerular corpuscles, Bowman’s capsule, and Bowman’s space were evident. The tubules retained their brush borders, but there was mild dilation of lymphatics and a slight infiltration of leukocytic cells into the interstitial tissue. Figure 5E,e depicted the kidney tissue from the Azil+GM group, where normal glomerular corpuscles were present along with hypertrophy of the epithelial lining of Bowman’s capsule and dilated Bowman’s space. The tubules maintained their brush borders, but the lymphatics were dilated, and there was edema with some leukocytic cell infiltration, and mild focal tubular hydropic degeneration, mildly congested blood vessels, and perivascular fibrosis with leukocytic infiltration.

Figure 5F, f depicted kidney tissue from the Para+SIS3+GM group, which displayed a notably improved histological appearance compared to the GM group. Normal glomerular corpuscles, Bowman’s capsule, and slightly dilated Bowman’s space were observed, and tubules maintained their brush borders with mild hydropic degeneration in some tubules. Figure 5G,g illustrated a section from the Azil+SIS3+GM group. This section showed normal glomerular corpuscles, Bowman’s capsule, and slightly dilated Bowman’s space, the tubules retained their brush borders, but there was moderate dilation of lymphatics, mild congestion of blood vessels, and perivascular edema.

### Immunohistochemical results of connective tissue growth factor

**Fig. 6:**
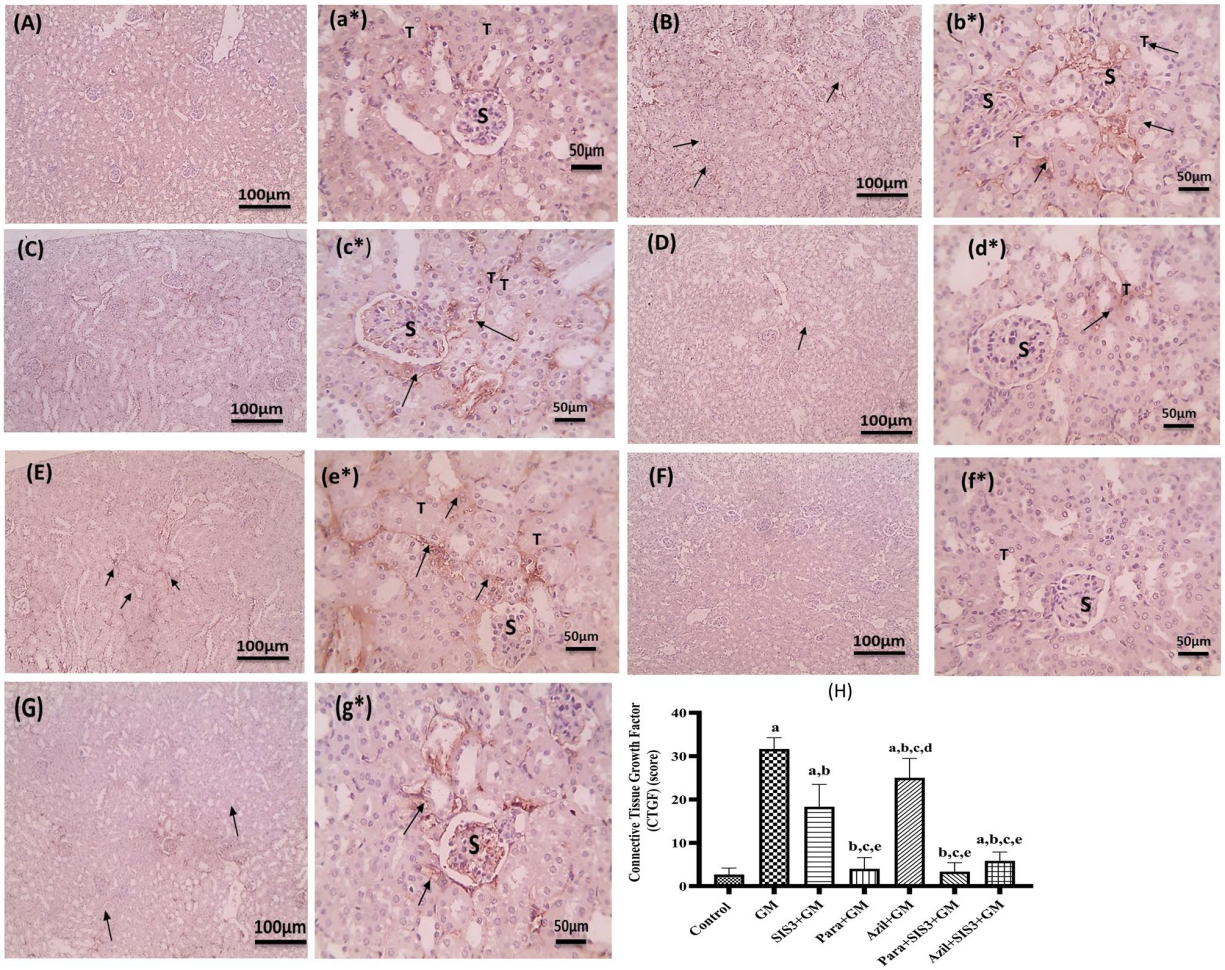
Sections of kidney tissues immunostained for CTGF. Magnification X: 100 bar 100 (large letter) and X: 400 bar 50 (small letter*). (A, a*): Control group, (B, b*): GM group, (C, c*): SIS3 group, (D, d*): Para group, (E, e*): Azil group, (F, f*): Para+SIS3+GM group, (G, g*): Azil+ SIS3+GM group. Connective tissue growth factor (CTGF) immune expression was indicated by brown color and was quantified using the color image analysis equipment (Leica Qwin 500 image analyzer coupled to a Leica microscope) at a magnification of x400. S refers to glomerular corpuscles and T refers to tubules. **H**: Relative expression of CTGF in renal tissues of the studied groups. The scores are the percentage of brown color relative to the blue background. Values are mean ± SD, significance was set at *p* < 0.05, *n* = 5. a: Significant vs control group, b: Significant vs GM group, c: Significant vs SIS3+GM, d: Significant vs Para+GM, e: Significant vs Azil+GM

Figure 6 illustrated the immunohistochemical staining of kidney sections for CTGF and the relative expression of CTGF in the studied groups. Control group exhibited a normal level of CTGF (2.67 ± 0.98). A marked positive brown tubular expression against CTGF was observed in the GM group highlighting significant increased expression of CTGF (31.67 ± 2.36). All, treated groups; SIS3+GM (18.33 ± 4.71), Para+GM (4 ± 2.30), Azil+GM (25 ± 4.08), and Para+SIS3+GM (3.33±1.89) and Azil+SIS3+GM (5.83 ± 1.86), showed significant decrease (*p* < 0.05) in CTGF expression compared to GM group. The least reduction was observed in Para+GM and Para+SIS3+GM groups, which showed normal level of CTGF expression compared to the control group.

### Immunohistochemical results of transforming growth factor-beta1

**Fig. 7:**
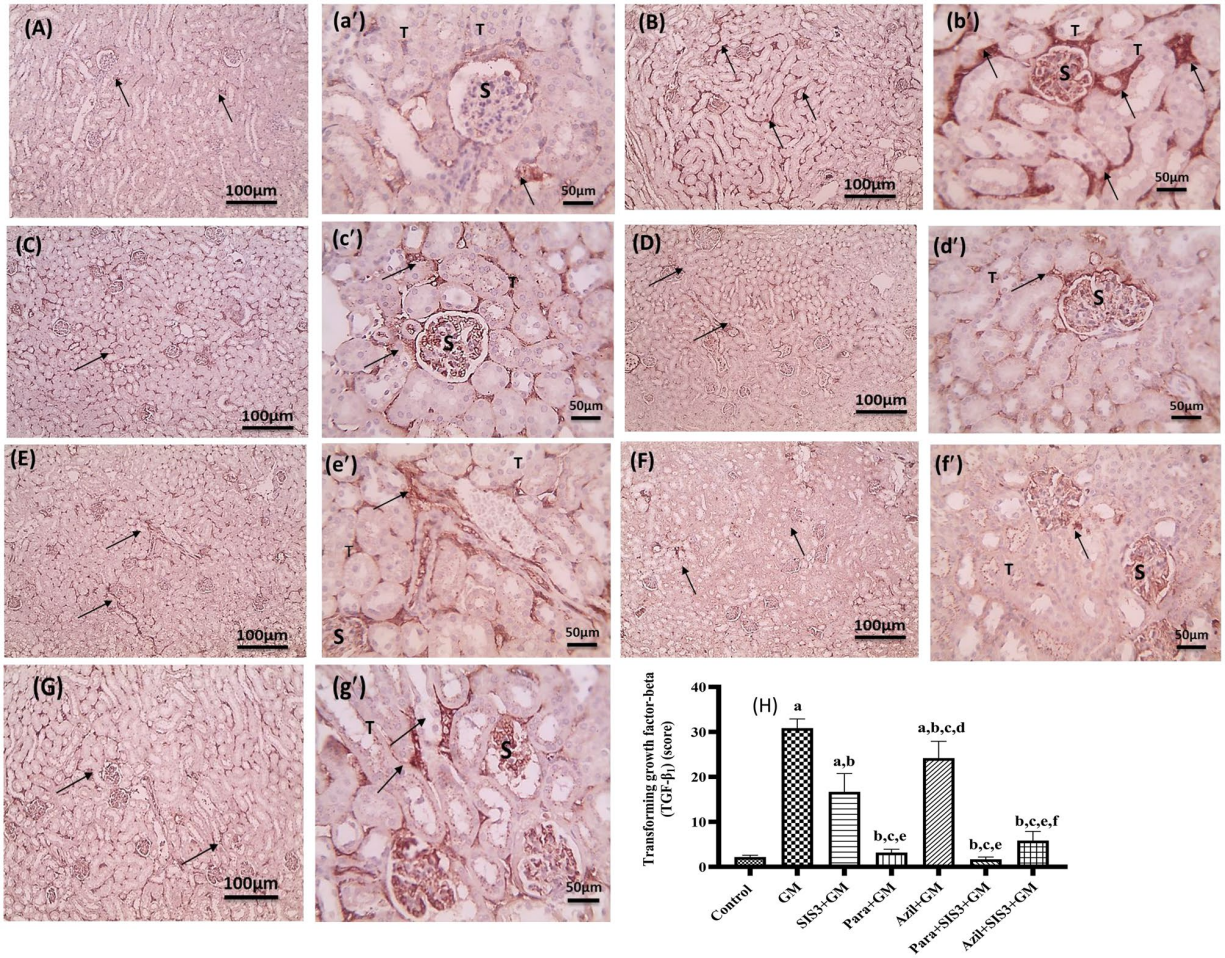
Sections of kidney tissues immunostained for TGF-β_1_. Magnifications X: 100 bar 100 (Capital letters) and X: 400 bar 50 (small letter*). (A, a*): control group, (B, b*): GM group, (C, c*): SIS3 group, (D, d*): Para group, (E, e*): Azil group, (F, f*): Para+SIS3+GM group, (G, g*): Azil+ SIS3+GM group. Transforming growth factor-beta1 (TGF-β_1_) immune expression was indicated by brown color and was quantified using the color image analysis equipment (Leica Qwin 500 image analyzer coupled to a Leica microscope) at a magnification of x400. S refers to glomerular corpuscles and T refers to tubules. **H**: Relative expression % of TGF-β_1_ in renal tissues of the studied groups. The scores are the percentage of brown color relative to the blue background. Values are mean ± SD, significance was set at *p* < 0.05, *n* = 5. a: Significant vs control group, b: Significant vs GM group, c: Significant vs SIS3+GM, d: Significant vs Para+GM, e: Significant vs Azil+GM, f: Significant vs Para+SIS3+GM

Figure 7 displayed the immunohistochemical staining of kidney sections for TGF-β_1_ and the relative expression of TGF-β_1_ in the studied groups. Control group indicated baseline levels of TGF-β_1_ (2.17 ± 0.37). A marked positive brown interstitial expression was observed in the GM group (30.83 ± 1.86), highlighting significant high levels of TGF-β_1_. However, treated groups; SIS3+GM (16.67 ± 3.73), Para+GM (3.17 ± 0.69), Azil+GM (24.17 ± 3.44), Para+SIS3+GM (1.67 ± 0.47), and Azil+SIS3+GM (5.83 ± 7.68) showed significant reduction (*p* < 0.05) in TGF-β_1_ expression. The least reduction in TGF-β_1_ was observed in Para+GM, Para+SIS3+GM, and Azil+SIS3+GM groups, which showed normal level of expression compared to the control group.

### Histopathological examination of renal fibrosis by Sirius red staining

**Fig. 8:**
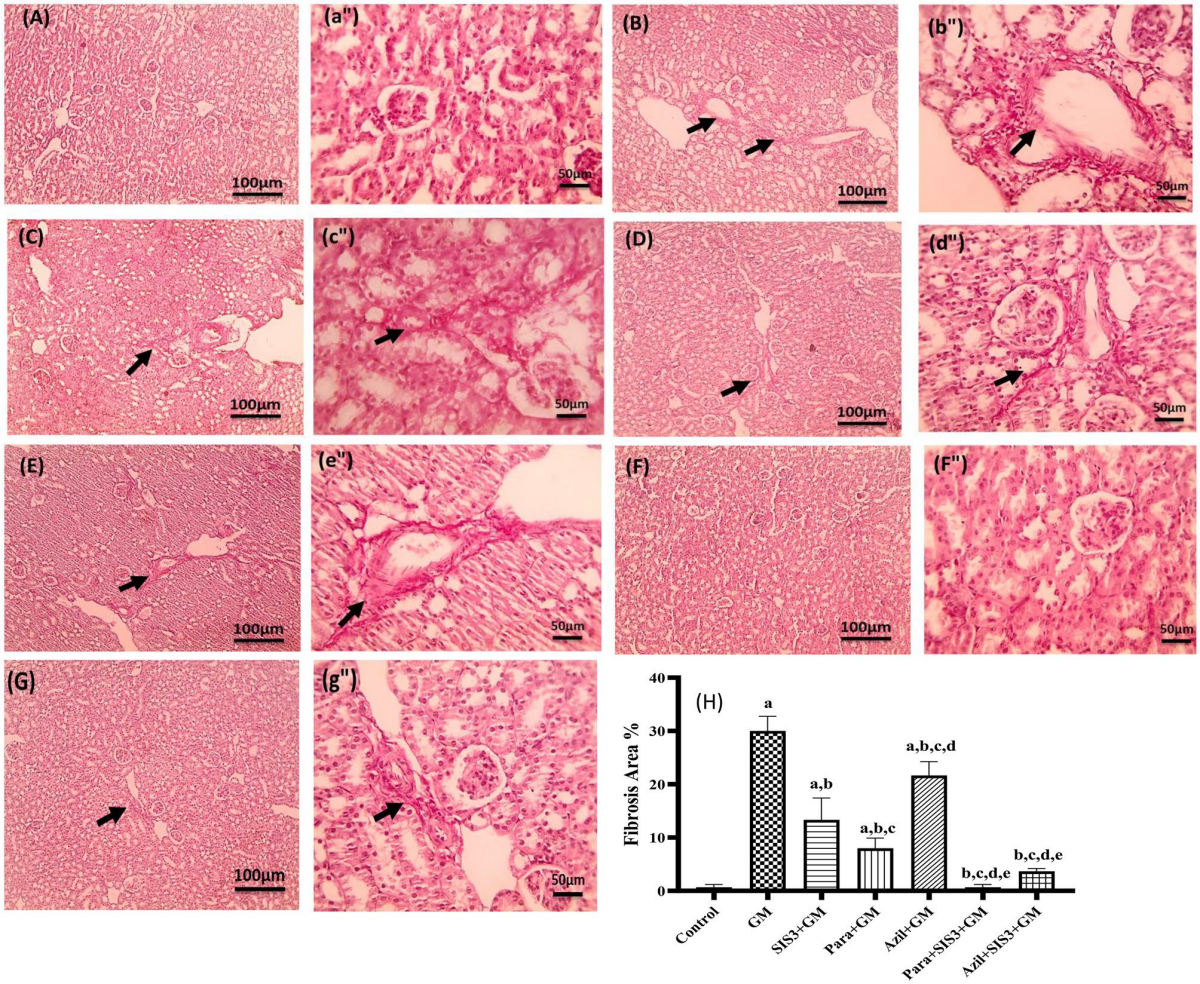
Effect of various treatments on kidney sections stained with Sirius Red in mice groups. Magnifications X: 100 bar 100 (Capital letters) and X: 400 bar 50 (small letter". (**A, a**"): Control group showed absent fibrosis, (**B, b**"): GM group showed marked interstitial red stained fibrous tissue (black arrow), (**C, c**"): SIS3 group showed more decrease in interstitial red stained fibrous tissue (black arrow) observed, (**D, d**"): Para group showed few red stained fibrous tissue strands (black arrow) observed in interstitial tissue , (**E, e**"): Azil group showed a slight decreased interstitial red staining of fibrous tissue (black arrow), (**F, f**"): Para+SIS3+GM group showed absent fibrosis , (**G, g**"): Azil+ SIS3+GM group showed very few red staining of fibrous tissue strands (black arrow) observed in the interstitial tissue. **H**: Relative expression of fibrosis area % stained with Sirius Red in renal tissues of the studied groups. The scores are expressed as the fibrosis area %. Values are mean + SD, significance was set at p < 0.05, n=5. a: Significant *vs* control group, b: Significant *vs* GM group, c: Significant *vs* SIS3+GM, d: Significant *vs* Para+GM, e: Significant *vs* Azil+GM, f: Significant *vs* Para+SIS3+GM.

Figure 8 showed kidney tissue sections from various experimental groups stained with Sirius red and the relative expression of Sirius red in the fibrotic tissue of the studied groups. Control group showed absent renal fibrosis (Fig. 8A,a”) (*p* < 0.05, 0.66 ± 0.51). In contrast, kidney tissue from GM group, revealed significant pathological changes; markedly interstitial red stained fibrous tissue (Fig. 8B,b”, H) (*p* < 0.05, 30.00 ± 2.76). Fig. 8C,c” showed kidney tissue from the SIS3+GM group, showed more decrease in interstitial red stained fibrous tissue (*p* < 0.05, 13.33 ± 4.08). Figure 8D,d” showed that in Para+GM group, few red stained fibrous tissue strands (*p* < 0.05, 8.00 ± 1.99). Fig. 8(8E,e” &H) depicted the kidney tissue from the Azil+GM group, showed slightly decreased interstitial red stained fibrous tissue (*p* < 0.05, 21.67 ± 2.58). Fig. 8(8F,f” & H) depicted kidney tissue from the Para+SIS3+GM group, showed absent fibrosis (*p* < 0.05, 0.66 ± 0.51). Fig. 8(8G,g” &H) illustrated a section from the Azil+SIS3+GM group, showed very few red stained fibrous tissue strands observed in the interstitial tissue (*p* < 0.05, 3.66 ± 0.51).

### Immunohistochemical analysis of Collagen 1 in the studied groups

**Fig. 9:**
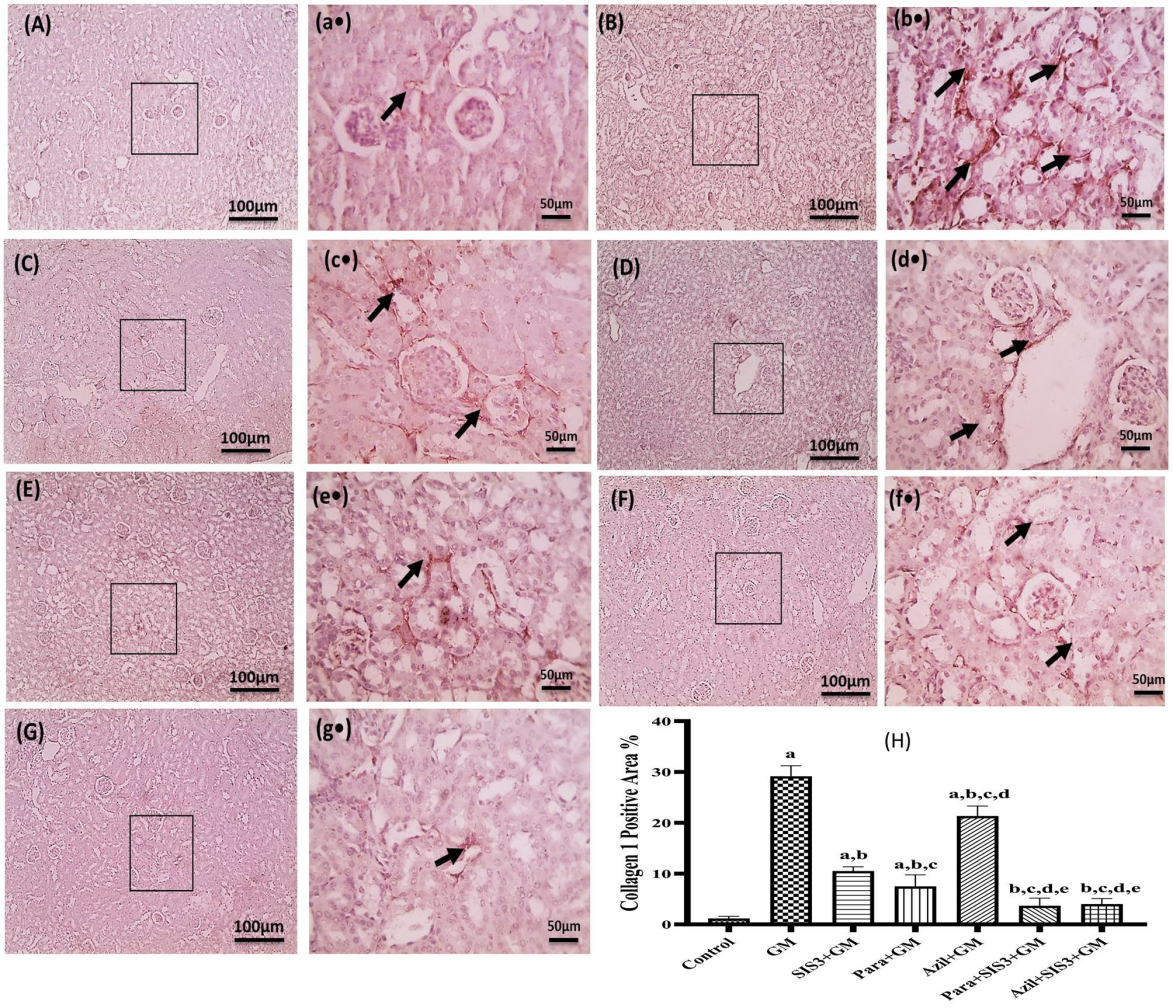
Effect of various treatments on immunohistochemical localization of type I collagen in kidney tissue of mice groups. Magnifications: magnifications X: 100 bar 100 (capital letter) and X: 400 bar 50 (small letter•). (**A, a**•): control group showed mild positive brown interstitial staining for collagen-1 (black arrow), (**B, b**•): GM group showed increased positive brown interstitial staining for collagen-1 (black arrow), (**C, c**•): SIS3 group, (**D, d**•): para group and (**E, e**•): azil group showed moderate decrease in positive brown interstitial staining for collagen-1 (black arrow), (**F, f**•): Para+SIS3+GM group &; (**G, g**•): Azil+ SIS3+GM group showed mild positive brown interstitial staining for collagen-1 (black arrow). **H**: The relative expression % of type I collagen immunohistochemical localization in renal tissues of studied groups. The scores are collagen-1 positive area%. Values are mean ± SD, significance was set at *p* < 0.05, *n* = 5. a: Significant vs control group, b: Significant vs GM group, c: Significant vs SIS3+GM, d: Significant vs Para+GM, e: Significant vs Azil+GM, f: Significant vs Para+SIS3+GM

Figure 9 illustrated the immunohistochemical staining of kidney sections for collagen 1and the relative expression of collagen 1 in the studied groups. Control group exhibited a mild positive brown interstitial staining (1.16 ± 0.41). A marked increase with positive brown interstitial staining was observed in the GM group highlighting significant increased expression of collagen 1(29.16 ± 2.04). All, treated groups; SIS3+GM (10.50 ± 0.84), Para+GM (7.5 ± 2.26), Azil+GM (21.33 ± 1.96), and Para+SIS3+GM (3.66±1.51) and Azil+SIS3+GM (4.00 ± 1.09), showed significant decrease (*p* < 0.05) in collagen 1 expression compared to GM group. The least reduction was observed in Para+SIS3+GM groups, which showed normal level of collagen 1 expression compared to the control group.

## Discussion

Acute kidney injury (AKI) is characterized by an abrupt decline in kidney function [43] which can be caused by toxins, drugs, infections, or inflammatory illnesses that harm the kidney [44]. AKI can lead to renal fibrosis, in which TGF-β_1_ is playing a significant role [45]. Therefore, the present study aimed to investigate the possible antifibrotic effect of paraxanthine and azilsartan in GM-induced kidney fibrois, and whether it could be mediated by modulation of TGF-β_1_ /Smad pathway and miRNA-21/miRNA-200b expression.

GM was selected because it is a well-established and widely accepted experimental model of nephrotoxicity and renal fibrosis, closely mimicking key pathological features observed in chronic kidney injury, including tubular damage, inflammation, oxidative stress, and fibrotic remodeling [46]. Moreover, gentamicin-induced nephropathy is strongly associated with activation of the TGF-β1/Smad signaling pathway, making it particularly suitable for mechanistic studies targeting renal fibrosis [2, 3].

In the present study, GM-induced nephrotoxicity was evidenced by histopathological findings as demonstrated by significant injury, including blood vessel congestion, lymphatic dilation, leukocyte infiltration, fibrosis, edema, loss of tubular brush borders and severe cell degeneration. In addition, the blood and urinary kidney function biomarkers indicated severe damage. These findings were in line with Gamman et al., 2023 [47].

The current treatment with Para or Azil alleviated renal fibrosis and retained the histological architecture, where the improvement in Para treated group was more evident than in Azil treated group. Moreover, both Para and Azil monotreatments partially corrected the blood and urine biochemical measurents. Our results were consistent with other previous studies which reported that administration of paraxanthine maintained BUN and creatinine near to normal level in mice [17], and that Azil treatment to nephrotoxic rats improved kidney function and decreased BUN and serum creatinine [22, 48].

In renal fibrosis, TGF-β_1_ is the most effective inducer of EMT, stimulating the production of ECM proteins such as collagen I and fibronectin [49]. Mice lacking Smad3 expression, when exposed to unilateral ureteral obstruction, showed delayed renal fibrosis, EMT, monocyte influx and collagen deposition [50]. We assumed that the beneficial role of Para and Azil could be mediated by inhibition of TGF-β_1_/Smad pathway, therefore we utilized Smad3 inhibitor (SIS3) either alone or in combination with Para or Azil.

Azilsartan was specifically chosen due to its higher affinity and longer-lasting blockade of the angiotensin II type 1 receptor compared with other ARBs. Additionally, azilsartan has been reported to exert pleiotropic renoprotective and anti-fibrotic effects, including attenuation of oxidative stress, inflammation, and TGF-β–mediated fibrotic signaling, beyond its antihypertensive action [48, 51].

The present investigation demonstrated that treatment with SIS3 reversed the abnormal expression of Smad3, Smad 2 & Smad 7 due to GM exposure. The upregulation of Smad7 by SIS3 treatment could be attributed to the ability of SIS3 to inhibit the Smad3-Smurf2-mediated degradation of Smad7, thereby preventing renal Smad7 degradation and suppressing NF-κB-dependent renal inflammation [52].

Both Para & Azil, in the current study, showed decreased levels of Smad2 & Smad3 expression but enhanced the expression of Smad7, in GM-intoxicated mice, similar to SIS3. Moreover, when SIS3 was combined with Azil, it showed a more potentiating effect towards increasing the expression of Smad7, but insignificant change in Smad3 expression compared to either treatment alone. Previous reports [48, 53] documented that azilsartan decreased Smad2/3 expression in the rat kidney tissue exposed to cisplatin and the expression of Smad7 was significantly increased in liver fibrosis of rats treated with perindopril or valsartan. On the other hand, combing SIS3 with Para exhibited a significant reduction in Smad2& Smad3 expression *versus* SIS3 and Para individual treatments, but this combination was less effective regarding Smad7 expression. Paraxanthine is a primary metabolite of caffeine in humans [17]. These results were consistent with those of Gressner et al., 2010 [54] who demonstrated that caffeine increases intracellular cAMP which activates ubiquitin–proteasome pathways. Thus, Smad2 is strongly degraded *via* Smurf2 than Smad3 as Smurf2 has high sensitivity toward both Smad2 and the TGF-β receptor complex (TGF-βRI/ALK5).

Our findings were further supported by the observed inhibition of TGF-β_1_ expression in kidney tissues of treated mice. Para significantly reduced TGF-β_1_ expression compared to GM group. These results were in accordance with Klemmer et al., 2011 [55] who reported that paraxanthine was found to be the most effective inhibitor of profibrogenic TGF-β in primary rat hepatocytes among caffeine and its three main demethylated metabolites.

Furthermore, Azil-induced decrease in TGF-β_1_ expression could be explained on the basis that as an angiotensin II receptor blocker, Azil can downregulate TGF-β_1_ expression, likely by inhibiting Ang II–mediated fibrotic pathways. This observation was in line with the results reported by Alaaeldin et al., 2023 [22]. Notably, the Para treated groups exhibited a more pronounced inhibition of TGF-β_1_ expression, compared to Azil treated groups. However, the inhibition of Smad3 by SIS3 potentiated the antifibrotic effects of both Para and Azil, as indicated by the greater inhibition of TGF-β_1_ in the combination treated group *versus* monotreated groups.

In the present study, CTGF was highly upregulated in the GM group, as evidenced by strong positive brown tubular immunostaining. Treatment with SIS3 led to a notable reduction in CTGF levels, likely due to its direct inhibition of Smad3 signaling. These results were consistent with previous findings, in which SIS3 has been reported to inhibit CXCL12, a signaling molecule involved in immune response and cell migration through promoting CTGF production in human lung fibroblasts [56, 57].

Importantly, the current investigation demonstrated the anti-fibrotic potential of both Azil and Para through inhibition of CTGF in renal tissues. Caffeine has been demonstrated to elevate cAMP levels, thereby activating protein kinase A (PKA), which in turn stimulated the cAMP response element-binding protein (CREB) and peroxisome proliferator-activated receptor gamma (PPAR-γ). Caffeine- induced activation of this PKA/CREB/PPAR-γ pathway resulted in pronounced inhibition of CTGF [56]. Moreover, caffeine was found to enhance Smurf2 activity, promoting further degradation of Smad2/3 and subsequently reducing both TGF-β_1_ and CTGF signaling [55]. This suggestion highlighted the results obtained by combined treatment with SIS3 and Para, which exhibited the greatest inhibition of CTGF expression among all treated groups. Similar results were obtained, herein, by Azil treatment or its combination with SIS3 significantly reduced CTGF expression *versus* GM group. Sukumaran et al., 2017 [58], illustrated that azilsartan with 1 or 3 mg tended to decrease the expression of CTGF in mice with diabetic cardiomyopathy.

The current investigation were further investigated *via* assessing renal fibrosis at the molecular level using Sirius Red staining histologically and collagen I immunohistochemistry. GM group showed significant interstitial collagen deposition, indicating the formation of renal fibrosis compared with control group. These results were consistent with those of Huang *et al.,* 2020 [59].

On the other hand, treatment with SIS3, Para, Azil, and their combinations greatly decreased renal fibrosis. Sirius Red staining and collagen I expression were restored to levels similar to the control group by the Para+SIS3 combination, which had the most noticeable impact of all the therapies. These results were consistent with those of Klemmer *et al.,* 2011 [55], who showed that paraxanthine reduced fibrosis and collagen buildup by reducing Sirius Red staining in liver histology and inhibition of profibrotic signaling.

While angiotensin receptor blockage can lessen fibrosis, targeting the Smad3 pathway may have a more direct and powerful antifibrotic impact. Azilsartan alone or combined with SIS3 also decreased collagen deposition, but to a lesser extent than Para+SIS3. The reduction in collagen I expression in treated groups demonstrates the agreement between present histological and molecular findings, which aligned with Reiss *et al.,* 2024 [60], they reported that Angiotensin receptor blockade has been shown to alleviate renal fibrosis by attenuating profibrotic signaling pathways and reducing interstitial collagen buildup.

Numerous miRNAs have been implicated in the development and progression of renal disorders [12]. In the current study, gentamicin (GM)-induced nephrotoxicity was associated with significant upregulation of miRNA-21 and downregulation of miRNA-200b compared to control group, which could be linked to the enhanced expression of TGF-β_1_ [14].

Our findings demonstrated that treatment with SIS3 reversed the abnormal expression of miRNA-21/200b due to GM exposure, which was consistent with the findings of Wu et al., 2022 [61], who reported that SIS3 inhibits Smad3 activation, thereby reducing the expression of pro-fibrotic miRNAs such as miRNA-21 and mitigating renal fibrotic responses. Another study demonstrated that SIS3 enhanced the protective effects of miRNA-200b/c in rat lung epithelial cells (RLE-6TN) by inhibiting the TGF-β_1_ /Smad3 pathway. miRNA-200b/c suppressed EMT and fibrosis by targeting the transcription factors ZEB1 and ZEB2 [62].

With reference to SIS3 group, herein, Para and Azil treatments produced comparable results. Both treatments contributed to miRNA-21 downregulation and miRNA-200b upregulation, suggesting a potential protective role against cellular stress in kidney injury models. Azil-induced downregulation of miRNA-21, could be accounted by the observed inhibition of TGF-β_1_, a known inducer of miRNA-21, in Azil treated groups. Previous studies have shown that azilsartan reduced inflammation and endothelial injury induced by oscillatory shear stress through the upregulation of kruppel-like factor 6 (KLF6), which in turn suppressed miRNA-21-induced IL-23 expression [63]. In the same context, the modulation of miRNA-21/200b by Para in the present work may be linked to the obtained suppression of TGF-β_1_/Smad3 expression. To our knowledge, this is the first study to evaluate the effect of Para on miRNA-21/200b expression.

## Conclusion

This study demonstrates that paraxanthine and azilsartan attenuate gentamicin-induced renal fibrosis in an experimental model. Both agents were associated with improved renal function and reduced histopathological damage. The observed antifibrotic effects appear to be mediated, at least in part, through modulation of the TGF-β1/Smad pathway and miRNA-21/200b signaling. These findings provide mechanistic insight and support further preclinical investigations to explore the antifibrotic potential of these agents.

## Limitation

Although the present findings demonstrate promising antifibrotic effects of paraxanthine and azilsartan in a gentamicin-induced renal fibrosis model, these results are limited to preclinical evidence. Further studies addressing pharmacokinetics, safety, and clinically relevant dosing are required before any conclusions regarding therapeutic or translational applicability can be drawn.

## Electronic supplementary material

Below is the link to the electronic supplementary material.

Supplementary Material 1

## Data Availability

Datasets and representative raw images are available upon reasonable request, and prepared in a public repository if required.
