# Supplementary figures and images for: Paraxanthine and azilsartan attenuate gentamicin-induced renal fibrosis via modulation of TGF-β1/Smad3/7 signaling and miRNA-21/miRNA-200b expression
Source: J Transl Med. 2026 May 11;24:684. doi: 10.1186/s12967-026-08051-y (PMC13182094; doi:10.1186/s12967-026-08051-y)

## Slide 1
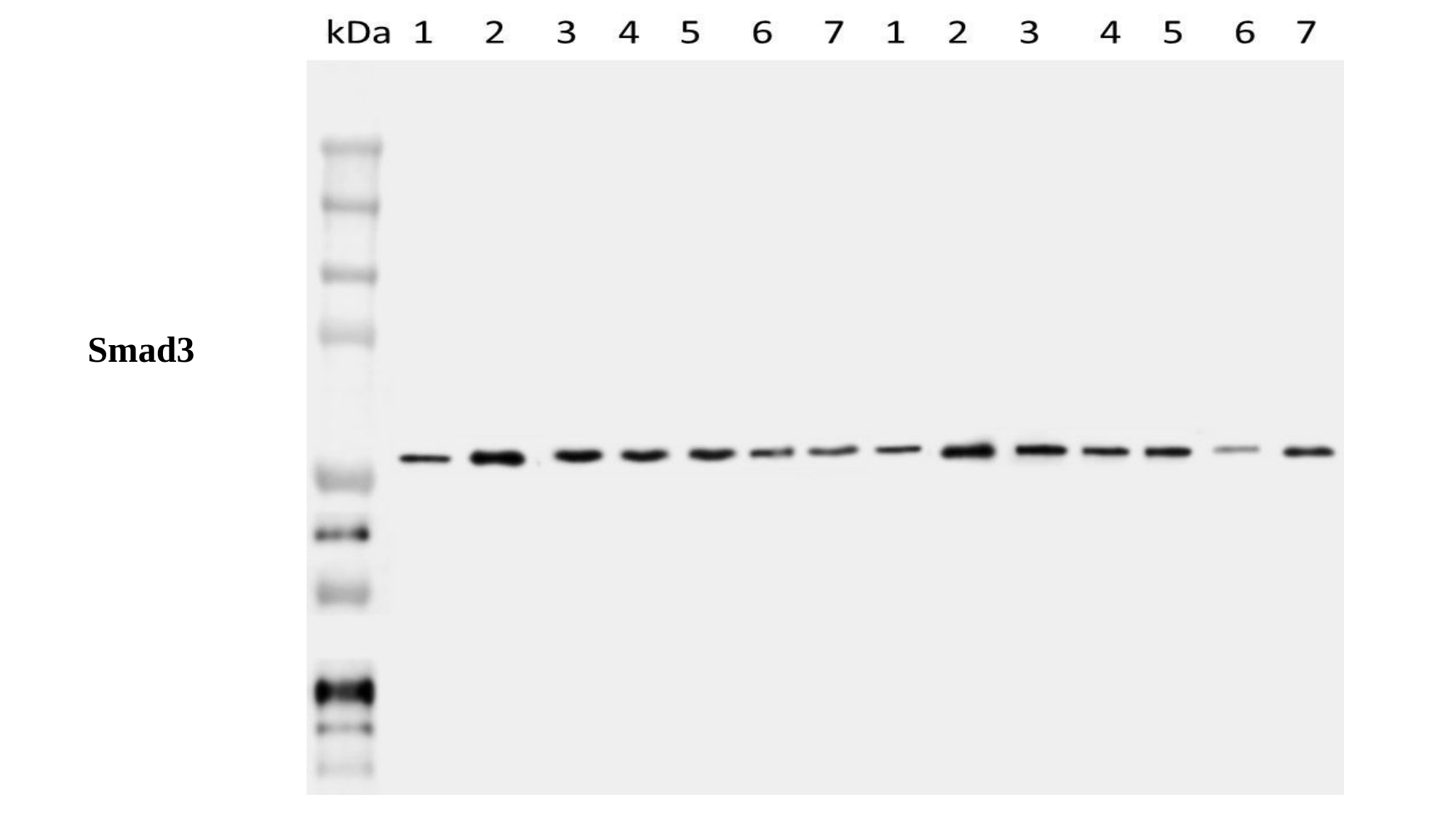

Smad3

## Slide 2
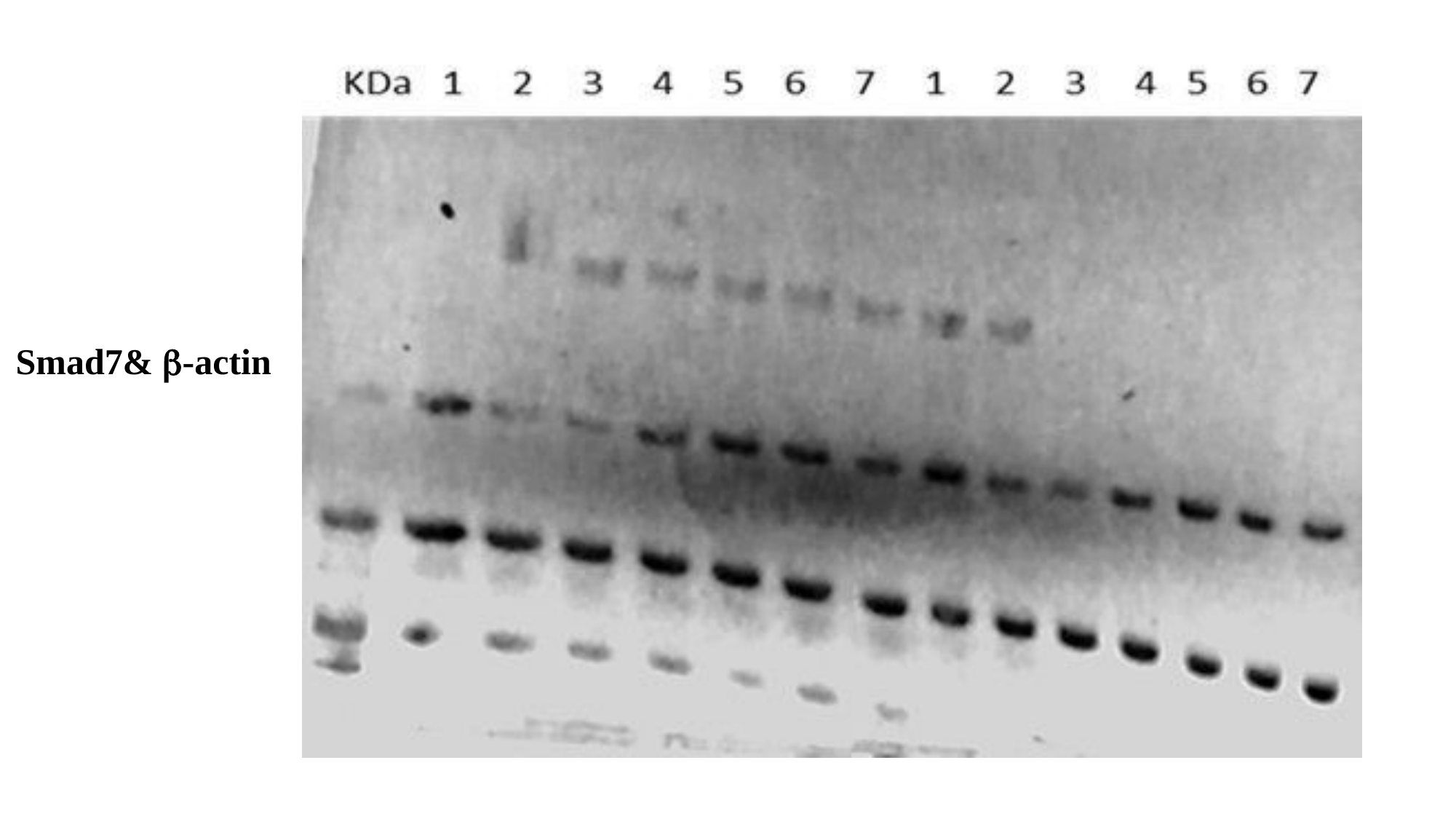

Smad7& β-actin

Supplement: Supplementary file 1 — Supplementary Material 1 [file 12967_2026_8051_MOESM1_ESM.pptx]
